# Modulating autophagy in *KRAS* mutant colorectal cancer using combination of oncolytic reovirus and carbamazepine

**DOI:** 10.1371/journal.pone.0326029

**Published:** 2025-06-17

**Authors:** Aaron Shaykevich, Danbee Chae, Isaac Silverman, Sanjay Goel, Radhashree Maitra

**Affiliations:** 1 Department of Biology, Yeshiva University, New York, New York, United States of America; 2 Rutgers Cancer Institute of New Jersey, New Brunswick, New Jersey, United States of America; The Third Affiliated Hospital of Sun Yat-Sen University, CHINA

## Abstract

Oncolytic reovirus is a potential therapeutic for colorectal cancer patients with a mutant *KRAS* gene. Reovirus hijacks the autophagic machinery and preferentially induces apoptosis in patients with a *KRAS* mutation. However, reovirus on its own is not currently a viable treatment and requires enhancement with combination therapies. Carbamazepine, an FDA-approved drug in use for epilepsy, is an autophagy inducer and is used in this study in conjunction with reovirus. The dual treatment was able to reduce cancer viability in mutated *KRAS* cell lines and was more effective than with reovirus alone. Carbamazepine and reovirus increased autophagy-related proteins and mRNA in mutant *KRAS* compared to wildtype *KRAS* which is crucial for autophagy-induced apoptosis. Transmission electron microscopy results show increased autophagosome formation in the combination therapy, as well as a decrease in condensed chromatin. The combination therapy effectively increased the apoptosis induced by reovirus alone and is a viable treatment for patients with a mutant *KRAS*.

## Introduction

Colorectal cancer (CRC) is among the most common cancer diagnoses. In the United States, CRC is the second most deadly cancer for both men and women [1]. Approximately 40%−50% of patients with CRC will be found to have a mutated *KRAS* gene, encoding the K-ras protein [2,3]. Among the most common mutations for *KRAS* is at the G12 or G13 residues. K-ras is a GTPase involved in signal transduction including the MAPK and PI3K signaling pathways [4,5]. When *KRAS* is mutated, many pathways are activated leading to unfettered cancerous cell growth [4–6]. Mutated *KRAS* also leads to the suppression of apoptosis and induction of autophagy [5]. Therefore, the presence of a mutated *KRAS* gene often leads to worse prognoses and outcomes in patients with CRC [7]. *KRAS*-inhibitors, such as Sotorasib and Adagrasib, are currently in use for patients with specific *KRAS* mutations [8,9]. These treatments are promising but at this point still require further study into additional pathways that can be targeted for optimal treatment. Furthermore, it has been demonstrated that patients may acquire resistance to these inhibitors [8,9]. Therefore, other methods of targeting *KRAS* are necessary for effective treatment in patients with CRC.

Autophagy, a process promoting cell survival by recycling damaged cell components, can sometimes act as a double-edged sword, having both the ability to promote cancer cell survival and induce apoptosis [10–12]. The mechanism by which autophagy induces apoptosis is highly specific and is often a cell stress response. Autophagy, combined with cell stress such as cell starvation, is thus a promising method for targeting cancer. Oncolytic Reovirus (pelareorep, referred to as REO in this paper) is a nonenveloped double-stranded RNA virus that selectively targets tumor cells [13]. In CRC, REO has been found to preferentially induce apoptosis in *KRAS* mutant (mut) CRC compared to in *KRAS* wild type (wt) [14]. Furthermore, it has been seen that REO involves the autophagic pathways in its mechanism of *KRAS*-mut killing [15]. REO on its own is a viable treatment for *KRAS*-mut CRC, and, in conjunction with an autophagy-inducing drug, may be a more effective method for treating *KRAS*-mut CRC.

One treatment we aim to study in conjunction with REO is carbamazepine (referred to as CBZ in this paper). Currently, CBZ is used to treat patients with epilepsy [16]. However, CBZ has also been seen to influence various pathways such as autophagy [17,18] and apoptosis. [19,20] In cancer specifically, carbamazepine has been found to work alongside *KRAS* to influence the expression of the SWI/SNF protein BRG1 [21], as well as induce apoptosis via VEGF and beta-catenin levels [22] and ROS [20]. Few studies have attempted to understand the mechanism behind carbamazepine-induced autophagy, however it is understood that one mechanism by which CBZ induces autophagy is via decreased IP3 signaling, leading to AMPK and ULK1 activity [17].

The aim of this study is to determine whether oncolytic reovirus-mediated cell death can be enhanced by the addition of carbamazepine. Autophagy, the cellular process of recycling organelles and proteins, is an established pathway reovirus utilizes to kill *KRAS*-mut cancer cells. We hypothesize that the addition of autophagy inducer carbamazepine can assist oncolytic reovirus in further killing *KRAS*-mut CRC.

## Methods

### Cell lines

Four CRC cell lines were used in this study: HCT116, HKe-3, SW620, and LIM2405. HCT116 (RRID:CVCL_0291) has a G13D *KRAS* mutation and SW620 (RRID:CVCL_0547) has a G12V *KRAS* mutation. HKe-3 (RRID:CVCL_9796) and LIM2405 (RRID:CVCL_4437) are *KRAS* wt. The HKe-3 and LIM2405 cell lines were obtained from Dr. Takehiko Sasazuki, Medical Institute of Bioregulation, Kyushu University, and Dr. Robert Whitehead, Ludwig Institute for Cancer Research, respectively. The HCT116 and SW620 cell lines were purchased from the American Type Culture Collection (ATCC®). All of the cell lines used were authenticated at the Albert Einstein College of Medicine Genomic Core Facility by short tandem repeat profiling.

### Cell culture

Cell lines were cultured in Roswell Park Memorial Institute (RPMI) 1640 media (Gibco™, Catalog #: 11875093), with 10% Fetal Bovine Serum (GemCell™, Catalog #: 100–500), 1% Non-Essential Amino Acids (Gibco™, Catalog #: 11140050), 2% HEPES buffer (Gibco™, Catalog #: 15630080), 1% Antibiotic-Antimycotic (Gibco™, Catalog #: 15240062), and 0.4% gentamicin (Gibco™, Catalog #: 15710064). The cells were maintained in an atmosphere of 5% CO2 at 37°C and passaged according to ATCC®’s recommended protocol.

### Carbamazepine (CBZ) preparation

CBZ powder (Supelco™, Catalog #: PHR1067-1G) was dissolved in absolute methanol at a concentration of 2 mg/ml (8.5mMol) and placed into single-use aliquots at −20°C. The final concentration the cells received of CBZ was 50uM, which was diluted at the time of treatment. CBZ is an FDA-approved treatment for epilepsy, and the concentration of 50uM is within the therapeutic reference range [23,24].

### Reovirus (REO) preparation

REO was provided by Oncolytics Biotech Inc and was stored for long term at −80°C and at +4°C for up to two weeks when in use. It was used at a multiplicity of infection (MOI) of 5 and 25.

### Cell treatment

Cells were cultured until 70% confluency, trypsinized (Corning™, Catalog #: 25-053-CI), and spun into cell pellets. Cell counting was done by using the Countess™ II Automated Cell Counter (Invitrogen™, Catalog #: AMQAX1000) via the Trypan Blue solution (Sigma-Aldrich™, Catalog #: T8154) following the manufacturer’s protocol.

All cell lines were prepared in a 100 mm plate (Denville™) at five million cells per plate. The cells remained for 24 hours (h) in 9 mL of cell culture media. After adherence, a plate from each cell line was treated with either 1mL of media, 1mL of 500uM CBZ solution (final concentration in media = 50uM), 1mL of REO (5MOI), or 1mL of both treatments. At either 6h or 24h post-treatment, the cells were harvested, and a portion was set aside for both protein and RNA extraction. Approximately 25% of the cell pellet was set aside for RNA analysis, while 75% was set aside for protein analysis.

### Protein extraction

Each cell pellet was suspended in 250 uL of freeze-thaw lysis buffer containing 247.5 uL Cell Extraction Buffer (Invitrogen™, Catalog #: FNN0011) and 2.5 uL of Thermo Scientific™ Halt™ Protease and Phosphatase Inhibitor Cocktail EDTA-free (100X) (Thermo Scientific™, Catalog #: 78445). The cell pellets underwent freeze-thaw, and were dipped in liquid nitrogen for 10 seconds, allowed to thaw, and then vortexed. This was repeated three times. After the third freezing, cells were placed on ice for 30 minutes. Cell pellets were then spun in a microcentrifuge at 15,000 RPM for 45 min and 4 °C. The supernatants were then placed into single-use aliquots at −80 °C.

### Protein estimation

Protein was estimated by a Bradford assay by combining one part H_2_O and one part Bradford for a total of 1mL solution. 5 uL of the protein sample (or an additional 5uL Bradford for the plate blank) was then added and homogenized. 200 uL of this mixture was then plated in a 96-well plate (Corning™, Catalog #: 3598) with triplicates for each sample. The solutions, including either protein samples or BSA standards, was then estimated using a SpectraMax Mini Microplate reader (Molecular Devices™, Catalog #: 76640-506) at 595nm absorbance.

### Western blotting

From each sample, 40 ug of protein was used. The protein was prepared by combining one part protein and one part 2X laemilli sample buffer (Bio-Rad™, Catalog #: 1610737). This mix was then placed in boiling water for 10 minutes. Each sample was loaded in a 4–20% gel (Bio-Rad™, Catalog #: 4561094). 2 uL of Magic marker (Invitrogen™, Catalog #: LC5602) and 10 uL of protein ladder (Thermo Scientific™, Catalog #: 26616) was loaded as well. A wet transfer was done at 80mV for 1h to a nitrocellulose membrane (Bio-Rad™, Catalog #: 1620215). The nitrocellulose membrane was then blocked in 1% BSA in TBS for 1h and then remained in primary antibody overnight. The primary antibody for ATG5 was Proteintech™ ATG5 Monoclonal Antibody at a 1:1000 dilution (Proteintech, Catalog #: 66744-1-Ig, RRID:AB_2882092). The primary antibody for Beclin-1 was Proteintech™ Beclin-1 Monoclonal Antibody at a 1:1000 dilution (Proteintech, Catalog #: 66665-1-Ig, RRID:AB_2882020). The primary antibody for LC3-B was Invitrogen™ LC3B Recombinant Rabbit Monoclonal Antibody (JJ090-6) at a 1:1000 dilution (Thermo Fisher Scientific, Catalog #: MA5-42459, RRID:AB_2911600). The primary antibody for RICTOR was Invitrogen™ RICTOR Monoclonal Antibody (J.644.6) at a 1:1000 dilution (Thermo Fisher Scientific, Catalog #: MA5-15006, RRID:AB_10985089). The primary antibody for ULK1 was Invitrogen™ ULK1 Recombinant Rabbit Monoclonal Antibody (JA58-36) at a 1:1000 dilution (Thermo Fisher Scientific, Catalog #: MA5–32699, RRID:AB_2809976). The primary antibody for PIK3C3 was Invitrogen™ VPS34 Monoclonal Antibody (H.107.3) at a 1:1000 dilution (Thermo Fisher Scientific, Catalog #: MA5–14791, RRID:AB_10975346). The primary antibody for the housekeeping protein, Beta actin, was Invitrogen™ Beta Actin Monoclonal Antibody (8H10D10) (Thermo Fisher Scientific, Catalog #: MA5–15452, RRID:AB_11001306). Analysis of the antibody binding was done using the Pierce™ Fast Western Blot Kit (Thermo Scientific™, Catalog #: 35050). The raw western blot files can be seen in S1 Fig.

### RNA extraction and quantification

The Invitrogen™ PureLink™ RNA Mini Kit (Invitrogen™, Carlsbad, CA, USA, Catalog #: 12183018A) was used for extracting RNA from the cell pellet and was done using the manufacturer’s protocol. The purified RNA was then placed into single-use aliquots and stored at −80 °C. The concentration of the extracted RNA was quantified using a Thermo Scientific™ NanoDrop 1000 (Thermo Scientific™, Catalog #: 2353-30-0010). The 260/280 value of the RNA was checked, and the RNA was only kept if the value was between 1.9 and 2.1.

### cDNA synthesis

A total of 1.5 ug of the extracted and quantified RNA was synthesized into cDNA using the iScript Reverse Transcription Supermix for RT-qPCR (Bio-Rad™, Catalog #: 1708841) as per the manufacturer’s protocol. A T100™ Thermal Cycler (Bio-Rad™, Catalog #: 1861096) was used. Using a Thermo Scientific™ NanoDrop 1000, the cDNA was estimated and then diluted to approximately 25 ng/uL using DEPC-treated water (Thermo Scientific™, Catalog #: R0601). The cDNA was then stored at −20 °C.

### Quantitative polymerase chain reaction (qPCR)

All primers were purchased from Sigma-Aldrich™ (Easy Oligo). The primers arrived pre-diluted at 100 uM. The primers were made into single-use aliquots upon arrival and stored at −20 °C. The sequences of the primers used can be seen in S1 Table.

Primers were prepared for qPCR by adding 20uL of forward and reverse primer as well as 180 uL of Thermo Scientific™ DEPC treated water leading to a 5 uM final concentration for both forward and reverse primers. 1 uL of the prepared primer mix, 4 uL of cDNA, and 5 uL Applied Biosystems™ PowerUp™ SYBR™ Green Master Mix (Applied Biosystems™, Catalog #: A25918), were added to all wells of a qPCR tube set (Bio Molecular Systems™, Catalog #: 71-107). All reactions were prepared in triplicates. The final reaction mix contained a 500 nM concentration of each primer and 100 ng of cDNA. qPCR was run using the Quantabio™ Q cycler (Quantabio™, Catalog #: 95900-4C). The data analysis was performed by calculating fold change using ΔΔCT. ΔCT was determined by subtracting the GAPDH CT value of the sample from its target gene CT value. ΔΔCT was determined by subtracting the ΔCT of untreated cells from the ΔCT of treated. Fold change values were calculated using the formula FC = 2^(−ΔΔCT).

### Cell viability assay

HCT-116 and Hke3 cells were pipetted into the inner wells of a BrandTech® cellGrade™ 96-well plate (BrandTech®, Catalog #: 781968) at 10,000 cells in 100uL per well, as well as blanks with just RPMI. 200uL of PBS was pipetted into the surrounding wells to prevent any evaporation of sample media. The plate was then placed in an incubator at standard conditions for 24h. Following that, each row of cells was treated with 10uL of either RPMI, CBZ (50uM), REO (25MOI), or CBZ + REO. All samples were prepared in sextuplets using all six inner wells per column. The 96-well plates were then placed back into the incubator for 24h.

After 24h, 11uL of Invitrogen™ PrestoBlue™ HS Cell Viability Reagent (Invitrogen™, Catalog #: P50200) was added to the inner-wells. The plate was then allowed to remain in the incubator for an additional hour. Absorbance of the 96-well plate was done using a SpectraMax Mini Multi-mode Microplate reader. The wavelength for detection was 570nm and normalized to 600nm.

The mean absorbance of RPMI-only wells was subtracted from the samples. The percent change between the untreated and the treated cells was then calculated. The percentage change between treated and untreated cells demonstrates the altered viability of cells post-treatment.

### Annexin V

24h after treatment (protocol described previously), an annexin V assay was performed on HCT-116 cells using the Annexin V-FITC Apoptosis Detection Kit (Sigma-Aldrich™, Catalog #: APOAF) as per the manufacturer’s protocol. The samples were run and analyzed in a Guava® EasyCyte™ mini. Cells were appropriately gated for apoptosis and were uniform across all samples. The data analysis of cellular apoptosis calculated the ΔAnnexin V+ cells by subtracting the percent of Annexin V+ cells in the control sample from the treated sample.

### Transmission electron microscopy (TEM)

HCT116 and HKe-3 cells were cultured until 70% confluency and then trypsinized. One million cells were plated in a 60 mm plate (Denville™, Catalog#: T1106). After 24h, one plate from each cell line was treated with either, RPMI, CBZ (50uM), REO (25MOI), or CBZ + REO. 9h later, monolayer cells were fixed in 0.1 M sodium cacodylate buffer with 2.5% glutaraldehyde. The cells were postfixed with 1% osmium tetroxide followed by 2% uranyl acetate and dehydrated through a graded series of ethanol. Samples were lifted with propylene oxide and embedded as a loose pellet in LX-112 resin (LADD Research Industries, Burlington VT). Using a Leica Ultracut UC7, ultrathin sections were cut and stained with uranyl acetate followed by lead citrate. Samples were then viewed on a JEOL 1400Plus transmission electron microscope at 120kV.

### Identification and measurements of cellular components in TEM photographs

TEM images were taken of cells in each treatment condition randomly. Autophagosomes and autophagolysosomes were manually identified and quantified at photos in 5000X magnification. CellProfiler (RRID:SCR_007358) was used for chromatin analysis. CellProfiler is an image processing software capable of deriving data from biological sample images [25]. CellProfiler contains modules with different functions which can be compiled to generate custom pipelines designed to analyze different components of the input image. We designed a pipeline to measure the area of the nuclei and the condensed chromatin within 1000X magnification TEM cell images in order to compare the treatments’ effects on cell morphology. Three modules were included in our pipeline: IdentifyObjectsManually; FillObjects; MeasureImageAreaOccupied. IdentifyObjectsManually allowed for precise tracing of each cell’s nucleus and condensed chromatin within; each tracing was done separately. This identifies to the software the “objects” of interest in the input images. FillObjects filled the area traced “objects,” which was finally measured using MeasureImageAreaOccupied.

### Statistical analysis

Statistical analysis was performed using Microsoft™ Office Excel. A two-tailed two-sample t-test was used for comparing fold changes. Outliers were determined and removed using Iglewicz and Hoaglin’s outlier test with modified z-scores using the outlier criterion of a modified z-score ≥ 3.5.

## Results

### Oncolytic reovirus and carbamazepine increases autophagic protein expression in *KRAS*-mut

Previous studies have found that carbamazepine and oncolytic reovirus as single agents are capable of inducing autophagy in certain conditions [15,18]. We thus analyzed the expression of autophagic proteins ATG5, Beclin-1, LC3B, RICTOR, PIK3C3, and ULK1 following the dual treatment.

**Fig 1:**
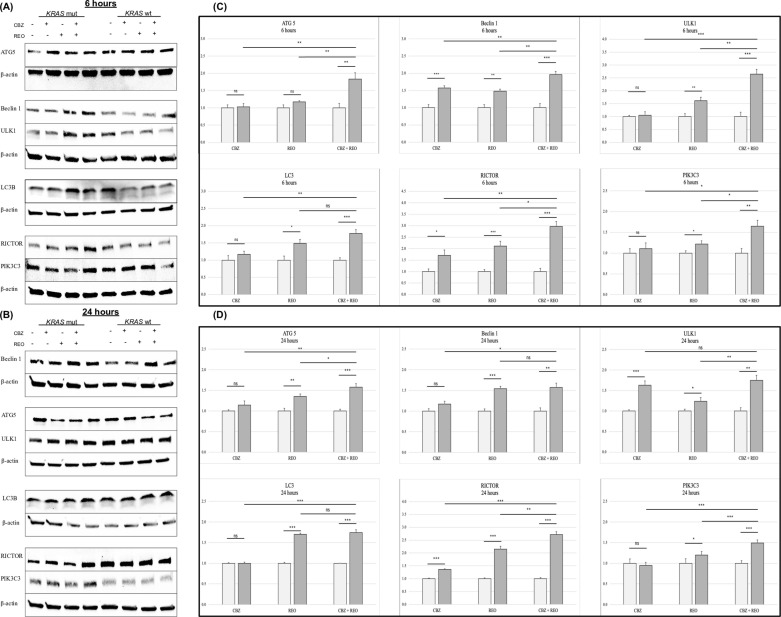
Western blot and densitometry of autophagy-related proteins. (A and B) Expression of autophagy-related proteins in *KRAS*-mut and *KRAS*-wt when untreated (-/-), CBZ treated (+/-), REO treated (-/+), and dual treated (+/+). (A) Representative blots of autophagy-related proteins ATG5, Beclin1, ULK1, LC3B, RICTOR, and PIK3C3 at 6h post-treatment. (B) Representative blots of autophagy-related proteins ATG5, Beclin1, ULK1, LC3B, RICTOR, and PIK3C3 at 24h post-treatment. (C and D) Significance indicated with asterisks (*). * indicates p < 0.05 ** indicates p < 0.01 and *** indicates p < 0.001. (C) Densitometry analysis of fold change of *KRAS*-mut normalized to *KRAS*-wt (n = 5) at 6h. (D) Densitometry analysis of fold change of *KRAS*-mut normalized to *KRAS*-wt (n = 5) at 24h.

Overall, at 6h, *KRAS*-mut cells treated with CBZ + REO generally saw an increase in expression compared to treated *KRAS*-wt cells (Fig 1A) which generally saw a decrease in expression compared to untreated. Similar trends were seen at 24h as well (Fig 1B).

At 6h (Fig 1C), *KRAS*-mut cells treated with CBZ did not express differently than *KRAS*-wt except in Beclin-1 and RICTOR (p < 0.001 and 0.05, respectively). *KRAS*-mut cells treated with REO increased expression for most proteins than *KRAS*-wt, with only ATG5 not reporting a significant difference. For *KRAS*-mut cells treated with both CBZ + REO, all proteins were significantly overexpressed. For almost all proteins, the individual treatments were significantly less effective at inducing autophagy when compared to the dual treatment. At 24h (Fig 1D), *KRAS*-mut cells treated with CBZ remained overexpressing RICTOR, but not Beclin-1, however, ULK1 was now additionally overexpressed (p < 0.001 for both). All proteins were upregulated in *KRAS*-mut cells treated with REO compared to *KRAS*-wt. For *KRAS*-mut cells treated with both CBZ + REO, all proteins were significantly overexpressed. At this time-point, half of the proteins were significantly overexpressed in the dual treatment when compared to the solo treatments.

### Transcription of autophagy-related mRNA are increased following a dual treatment

**Fig 2:**
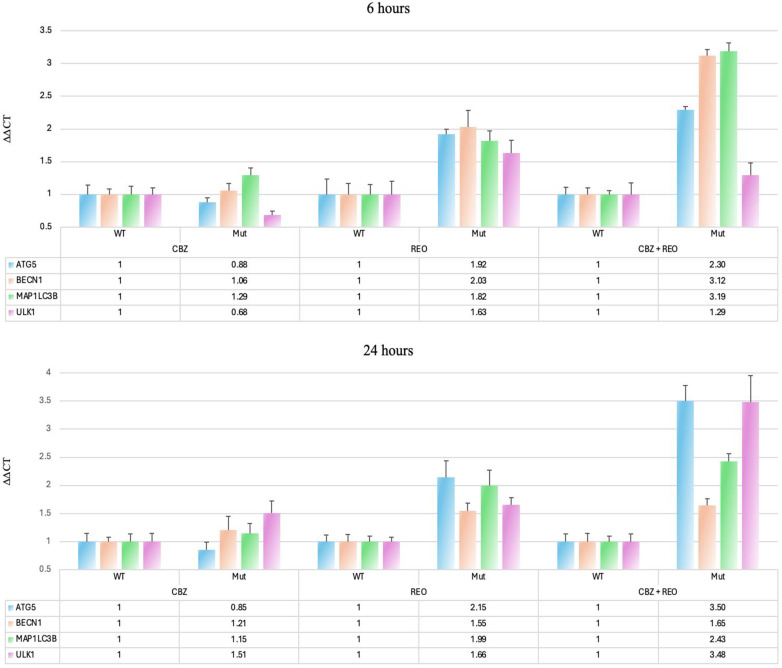
Expression of autophagy-related mRNA. ΔΔCT qPCR results of treated *KRAS*-mut, normalized to *KRAS*-wt (n = 5) at both 6 and 24h. Only *ULK1* at 6h was upregulated in CBZ in *KRAS*-mut. All genes were significantly upregulated in REO except *ATG5* and *ULK1* at 6h. The dual treatment significantly upregulated all genes at both time-points and further was significantly greater compared to REO for *ATG5* at both 6 and 24h (p < 0.01 for both), *BECN1* at 6h (p < 0.01), *MAP1LC3B* at 6h (p < 0.001), and ULK1 at 24h (p < 0.01).

In order to establish that the translation of autophagy-related proteins was due to an increase in expression, we sought to confirm that there was an elevated transcription of autophagy-related genes via mRNA expression (Fig 2).

At 6h, in *KRAS*-mut cells, mRNA expression of certain genes was altered in all treatments compared to *KRAS*-wt. For cells treated with CBZ, *ULK1* mRNA expression decreased (p < 0.05). *KRAS*-mut cells treated with REO saw an increase in *BECN1* (encoding Beclin-1) and *MAP1LC3B* (encoding LC3B) (p < 0.01 for both). The combination therapy was effective in significantly increasing *ATG5*, *BECN1*, and *MAP1LC3B* (p < 0.001 for all). Furthermore, the expression of *ATG5*, *BECN1*, and *MAP1LC3B* was significantly increased for *KRAS*-wt when compared to just REO (p < 0.01, p < 0.01, and p < 0.001, respectively). At 24h, for *KRAS*-mut cells treated with CBZ, there were no significant differences in *KRAS*-mut compared to *KRAS*-wt. *KRAS*-mut cells treated with REO saw an increase in all four genes tested, *ATG5*, *BECN1*, *MAP1LC3B*, and *ULK1* (p < 0.01, p < 0.05, p < 0.01, p < 0.01, respectively). The combination therapy, as well, was effective in significantly increasing *ATG5*, *BECN1*, *MAP1LC3B*, and *ULK1* (p < 0.001, p < 0.01, p < 0.001, p < 0.001, respectively). Furthermore, the expression of *ATG5*, *ULK1* was significantly increased for *KRAS*-wt when compared to just REO (p < 0.01 for both). This data shows that autophagy-related proteins and genes are indeed being overexpressed at both the protein and mRNA levels in the dual treatment.

### Dual treatment synergistically increases autophagosome formation and cell degradation

**Fig 3:**
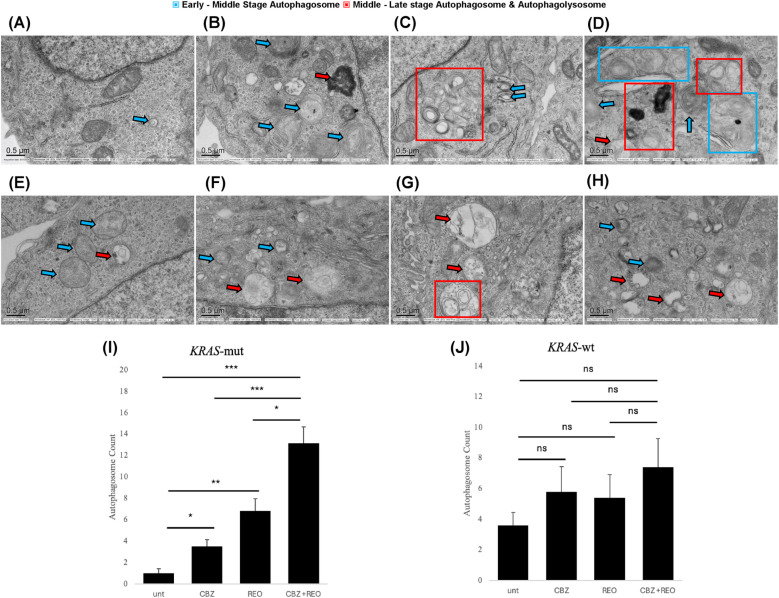
TEM of autophagosomes. (A) Representative image of a *KRAS*-mut cell, few autophagosomes are formed. (B) Representative image of a *KRAS*-mut cell treated with CBZ, some smaller autophagosomes. (C) Representative image of a *KRAS*-mut cell treated with REO, larger and complex autophagosomes and autophagolysosomes. (D) Representative image of a *KRAS*-mut cell treated with CBZ + REO, large number of autophagosomes and at different stages. (E) Representative image of a *KRAS*-wt cell, some autophagosomes are formed. (F) Representative image of a *KRAS*-wt cell treated with CBZ, some autophagosomes of varying size. (G) Representative image of a *KRAS*-wt cell treated with REO, few larger autophagosomes and autophagolysosomes. (H) Representative image of a *KRAS*-wt cell treated with CBZ + REO, some autophagosomes of varying size. (I and J) Significance indicated with asterisks (*). * indicates p < 0.05 ** indicates p < 0.01 and *** indicates p < 0.001. (I) Average of four pictures per sample. *KRAS*-mut cells treated with CBZ, REO, and CBZ + REO all had increased autophagosome formation (p < 0.05, p < 0.01, p < 0.001, respectively). CBZ + REO treatment saw more autophagosomes than the individual CBZ or REO treatment (p < 0.001 and p < 0.05, respectively). (J) Average of four pictures per sample. No significant difference in autophagosome number across all treatments.

In order to confirm that autophagy is induced by CBZ, REO, and the dual treatment, transmission electron microscopy (TEM) was used to visualize autophagosomes. Few autophagosomes were seen in *KRAS*-mut CRC (Fig 3A). *KRAS*-mut cells treated with CBZ (Fig 3B) or REO (Fig 3C) had an increase in the average number of autophagosomes (p < 0.05 and p < 0.01, respectively). *KRAS*-mut cells treated with a dual treatment (Fig 3D) had significantly more autophagy compared to the individual CBZ or REO treatments (p < 0.001 and p < 0.05, respectively). Few autophagosomes were found in *KRAS*-wt cells (Fig 3E), and the increase in autophagosomes was not significant in the CBZ treatment (Fig 3F), REO treatment (3G), and dual treatment (Fig 3H). When combining CBZ and REO, the effect on *KRAS*-mut cells is additive, as the sum of both the individual treatments is still less than the dual treatment (Fig 3I). The same cannot be said for *KRAS*-wt, where both treatments do appear to have an effect, but the change cannot be reported as significant, and the dual treatment does not appear additive (Fig 3J).

### Carbamazepine enhances oncolytic reovirus-mediated cell death and cell apoptosis in *KRAS*-mut CRC

**Fig 4:**
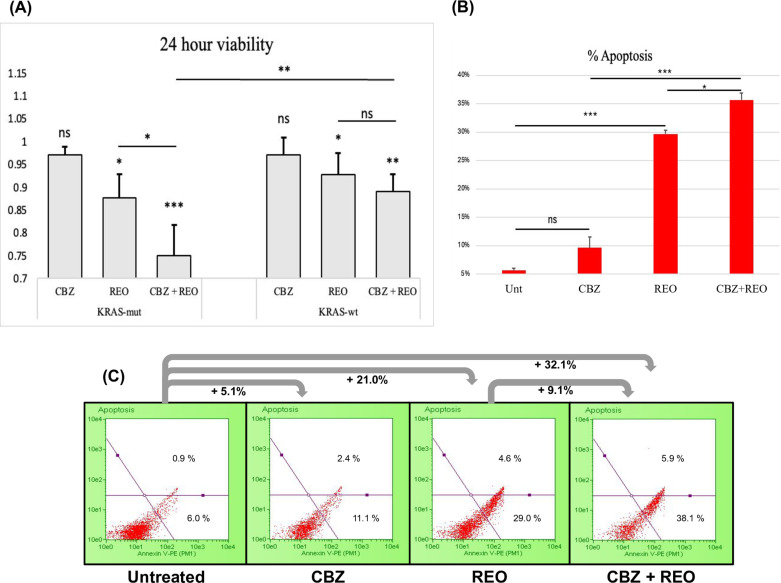
Cell viability and apoptosis detection. (A) Cell viability at 24 (n = 4). Significance between REO and the dual treatment was seen in *KRAS*-mut but not *KRAS*-wt cells. (B) Quantified Annexin V assay results (n = 3) displaying that *KRAS*-mut cells treated with reovirus had an increase in apoptosis (p < 0.001) and that the dual treatment further increased apoptosis (p < 0.05). (C) Flow cytometry dot plot of Annexin V assay results. Significance indicated with asterisks (*). * indicates p < 0.05 ** indicates p < 0.01 and *** indicates p < 0.001.

Having seen the alterations to autophagy in the KRAS-mut CRC following the dual treatment, we sought to understand its impact on cell viability and whether autophagy-induced apoptosis occurred. Using PrestoBlue™ HS Cell Viability Reagent, we analyzed cell viability post-treatment. The dual treatment was significantly more effective than single agent REO in *KRAS*-mut cells (p < 0.05). Besides the dual treatment being more effective than REO in *KRAS*-mut, the dual treatment was also more effective in *KRAS*-mut than in *KRAS*-wt (p < 0.01) (Fig 4A). Additionally, we sought to investigate if there was an increase in apoptosis by these treatments using Annexin V (Fig 4B). *KRAS*-mut cells treated with REO were found to have elevated apoptosis by around 24% (Fig 4C) (p < 0.001). When combining REO with CBZ, there was significantly more apoptosis in the dual treatment than both compared to the individual REO and CBZ treatments, 6% more than REO and 26% more than CBZ (p < 0.05 and p < 0.001, respectively). The dual treatment seemed to represent an additive effect on apoptosis.

### Genomic structure altered in oncolytic reovirus-treated cells and enhanced by the addition of carbamazepine

**Fig 5:**
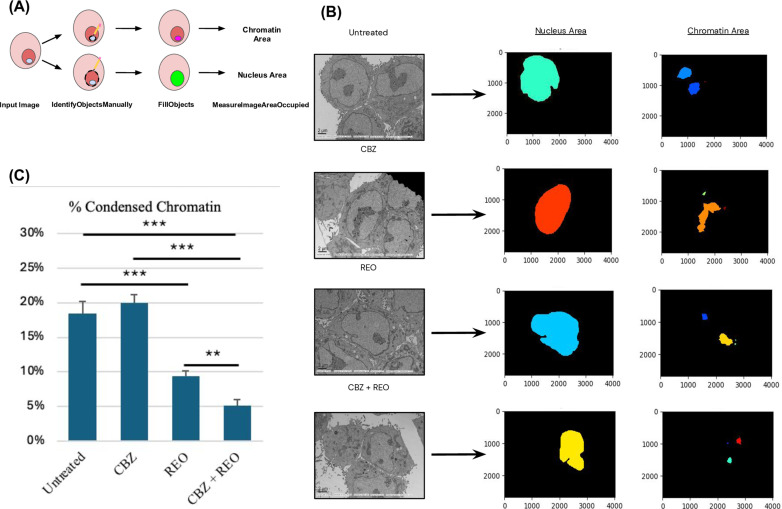
TEM indicating altered levels of condensed chromatin. (A) CellProfiler pipeline used to trace out and measure the area of the nuclei and chromatin of cells undergoing each treatment type (n = 4). (B) Representative 1000X TEM cell images of each treatment type with red arrows indicating condensed chromatin in the nuclei. CellProfiler processed images are to the right used to measure the areas of the nuclei and chromatin. (C) The areas of the nuclei and chromatin were calculated as a percentage using a ratio of each with the total image area. A second ratio was calculated between the chromatin ratio and the nucleus ratio of each cell to determine the proportion of condensed chromatin in each treatment (n = 4). Significance ranges indicated with asterisks (*). * indicates p < 0.05 ** indicates p < 0.01 and *** indicates p < 0.001.

After finding an increase of cell killing and autophagy in *KRAS*-mut CRC treated with the dual treatment, we sought to understand how the nucleus of the cells may act pre-apoptosis. The areas of the nucleus and condensed chromatin were measured using CellProfiler (Fig 5A).This was done for *KRAS*-mut cells with all treatment types (Fig 5B). There were significant differences between treatment type and chromatin area and subsequently the chromatin/nucleus ratio (S2 Table). The condensed chromatin in cells of each treatment type were found to exist either in one portion or multiple throughout the nucleus.

*KRAS*-mut cells treated with REO or CBZ + REO were found to have decreased condensed chromatin p < 0.001 for both) (Fig 5C). Cells treated with REO were found to have an altered chromatin structure, with condensed chromatin taking up nearly only half the area as in untreated cells at 9.41% compared to 16.94% in untreated (p < 0.001). Cells treated with the dual treatment had an enhanced effect of REO, altering chromatin condensation the most of the three treatments to 5.17% and significantly less compared to the single REO treatment (p < 0.01). In the dual treatment, the percentage area of chromatin condensation was decreased by over three times the percentage area in the untreated cells.

## Discussion

Oncolytic reovirus is emerging as a therapy targeting colorectal cancer [15,26]. However certain circumstances make the virus unable to be fully effective, such as an immunosuppressive tumor microenvironment [27]. Thus, the ability to add to the treatment of oncolytic reovirus with an additional treatment may enhance its ability to target cancer. Oncolytic reovirus has been seen to synergize with certain treatments, such as with the chemotherapy Irinotecan [14]. Previously, it has been seen that oncolytic reovirus preferentially targets mutant *KRAS* cells. The mechanism for this was highlighted as an altered induction in autophagy and thus autophagic cell death, depending on mutation status [15]. Therefore, we sought to understand how oncolytic reovirus may react with a simultaneous treatment of carbamazepine. Carbamazepine, a drug already in use for the treatment of epilepsy, has also been seen to interact with and induce autophagy [17].

We analyzed the expression of crucial proteins involved in the initiation and growth of autophagy and autophagosomes. The proteins analyzed successfully cover the various autophagic pathways and stages. ULK1 forms a complex that plays a role in autophagy initiation [28,29]. This complex helps initiate the binding of proteins in the Beclin-1 and PIK3C3 (VPS34) complex, which produces phospholipid PI3P, which begins the formation of the autophagosome [28]. ATG5 is a protein involved in the formation of the ATG5-ATG12-ATG16 complex which facilitates LC3 lipidation, thus leading to, and both are involved in, autophagosome growth [28,30]. RICTOR is a large component of the mTORC2 complex which phosphorylates Akt [31], and has been shown to have a correlation to autophagy expression in REO-treated cells [15]. RICTOR has also been seen to induce autophagy in certain conditions [32].

In this study, we found that reovirus increases ULK1 expression in mutant *KRAS* compared to wildtype *KRAS,* and that carbamazepine also increased ULK1 expression in mutant *KRAS* compared to wildtype *KRAS* at 24h. It has been seen in other studies that carbamazepine treatment activates ULK1 [17]. The dual treatment increased ULK1 levels much further at 6h compared to either of the individual treatments, however at 24h it expressed similarly to that of the individual carbamazepine treatment. ULK1 is directly regulated by the Ras/Raf pathway [33], of which *KRAS* is a crucial component, and thus it is likely that the treatment of carbamazepine on its own assists in *KRAS*-driven ULK1-initiated autophagy. Beclin-1 and PIK3C3 were both found overexpressed in reovirus-treated mutant *KRAS* at both 6h and 24h. When *KRAS* mutant cells were treated with just carbamazepine, only Beclin-1 was found overexpressed in *KRAS* mutant, and only at 6h. The dual treatment was effective at altering Beclin-1 and PIK3C3 expression at 6h, however not at 24h, where Beclin-1 was not significantly different than *KRAS* mutant cells treated with just reovirus. This suggests that at 6h when carbamazepine and reovirus individually were both effective, the impact on Beclin-1 expression was additive. When the *KRAS* mutation was not a factor in the Beclin-1 expression of carbamazepine-treated cells, reovirus was equally as effective as the dual treatment. The dual treatment also found that ATG5 had an altered expression much greater in *KRAS* mutant than in wildtype at both 6h and 24h. Finding ATG5 to be upregulated highly suggests that autophagy is elevated as overexpression of ATG5 is linked to increased autophagy [34].

RICTOR is one of the largest components of the mTORC2 complex. This complex is understudied compared to the similar mTORC1 complex [35,36]. One crucial factor of RICTOR is that in oncolytic reovirus-treated patients with a *KRAS* mutation, the expression of RICTOR increased alongside autophagy-related proteins [15]. In this study, we found that at both time-points, cells treated individually with either carbamazepine or reovirus were found to have a larger altered expression of RICTOR in mutant *KRAS* than in wild type. This suggests that RICTOR and the mTORC2 complex crosstalk with *KRAS*, and that increased autophagic signaling leads to higher RICTOR expression. Furthermore, the dual treatment significantly increases RICTOR expression further, significantly increasing compared to the individual treatments. This in turn leads to further increases in autophagy, as RICTOR is known to induce autophagy [32]. LC3B was found to be overexpressed in reovirus-treated *KRAS* mutant compared to wildtype but was not further enhanced in the dual treatment. However, it must be noted that LC3B-II is degraded during autophagy and the quantification of LC3 on its own is not indicative of autophagosome quantity [37,38]. The increased levels of other autophagy-related proteins indicate that autophagy was impacted by the dual treatment.

Following our findings that protein expression is altered in the presence of a *KRAS* mutation, we analyzed the mRNA expression of *ATG5*, *BECN1*, *MAP1LC3B*, and *ULK1.* It was discovered that at 6h, carbamazepine alone decreases *ULK1* in *KRAS* mutant relative to wildtype, which is consistent with previous findings [21]. Carbamazepine impacts the activity of many transcription factors [39], and in an environment of increased autophagy, it is likely that the transcription of *ULK1* is not needed, as *ULK1* is often transcribed when autophagy is necessary for cell survival [40]. When comparing *KRAS* mutant to wildtype, at 6h, oncolytic reovirus was effective at upregulating genes *ATG5*, *BECN1*, and *MAP1LC3B*, and at 24h, oncolytic reovirus was effective at upregulating all genes *ATG5*, *BECN1*, and *MAP1LC3B*, and *ULK1.* This has been found previously, where critical autophagy genes are upregulated post-reovirus treatment in mutant *KRAS* CRC [15]. Significantly, we found that the dual treatment was significantly more effective at upregulating *ATG5*, *BECN1*, and *MAP1LC3B* at 6h, as well as *ATG5* and *ULK1* at 24h. This emphasizes that carbamazepine enhances reovirus in initiating and expressing autophagy. The upregulation of these genes is critical in increasing overall autophagy and emphasizes how mutant *KRAS* is utilized to upregulate transcription factors [41].

Transmission electron microscopy, a method considered the gold standard for detecting and visualizing autophagosomes [42], was used to visualize the increase in autophagosomes post-treatment. In mutant *KRAS* cells, both carbamazepine and reovirus individually increased autophagosome formation. Visually, the autophagosomes in carbamazepine-treated cells appeared less mature and smaller in nature. In contrast, autophagosomes from reovirus were more mature and would target larger areas of the cell. When *KRAS* mutant cells were treated with both carbamazepine and reovirus, there was a larger amount and variety of autophagosomes present. The increase in the quantity of autophagosomes is also indicative of an increase in overall autophagy with the dual treatment. Furthermore, the size of the autophagosome can also affect which cargo is taken up by the autophagosome [43], and the wide variety of the dual treatment allows it to take up different types of components than the more selective individual treatments. In wildtype *KRAS*, the treatments impacted autophagosomes similarly to *KRAS* mutant in regard to appearance, however, it did not significantly alter the total amount of autophagosomes present.

Cell viability was tested to determine if carbamazepine enhances reovirus-mediated cell killing, and we found that this was the case for *KRAS* mutant cells. This showcases carbamazepine as a potential therapeutic in colorectal cancer, which has been seen previously, as carbamazepine alters levels of other crucial proteins [20–22]. We found that carbamazepine on its own was not capable of killing cancer cells beyond a small percentage, which has been previously reported [44]. However, this is not unanimous across the literature, with some research reporting *KRAS* mutant cells being killed with carbamazepine alone [22]. Annexin V was utilized at 24h to quantify the increase in apoptosis from treatment. Similar to our results of cell viability, carbamazepine did not significantly induce apoptosis. Cells treated with just oncolytic reovirus had a large increase in apoptosis and this was further increased by approximately with the addition of carbamazepine. Thus, the dual treatment is effective in inducing high levels of apoptosis in cancer.

Finally, using CellProfiler, the areas of the nucleus and condensed chromatin were measured. There were significant decreases in condensed chromatin in mutant *KRAS* cells treated with oncolytic reovirus and further decreases in cells treated with both oncolytic reovirus and carbamazepine. The results were consistent with previous studies that have distinguished apoptotic and autophagic conditions, characterizing autophagic cell death by either the lack of chromatin condensation or the process only occurring in its later stages [45,46]. Additionally, cell death-induced autophagy has been characterized by only moderate chromatin condensation [47]. In relation to reovirus infection, past studies have detected decreased levels of DNA synthesis initiation related to altered DNA supercoiling within the cell. This was found to be reversible by removing the nucleus from the infected environment [48]. Previous studies have also found that in cells undergoing REO-induced apoptosis an increase in condensed chromatin has been observed [49]. Additionally, CBZ has been observed to condense chromatin in some neuron cells [50], and a similar effect, yet not statistically significant, was observed in this study. A potential explanation for the altered levels of chromatin condensation in autophagy is the selective degradation of chromatin-modeling proteins, such as DNA methyltransferases and histone modifiers [51]. Epigenetic regulation of autophagy has recently been shown to involve such nuclear components. Thus, autophagy can effectively influence chromatin structure through reversible processes of epigenetics [52], which is supported by our findings.

## Conclusion

Finding an effective treatment for *KRAS* mutant colorectal cancer is of high priority. Oncolytic reovirus has been shown as one possible treatment, and its ability to preferentially target mutant *KRAS* is especially valuable. Reovirus has been shown to hijack autophagic machinery and has been tested both on cell lines and in patients [15]. We have identified carbamazepine as a drug that enhances reovirus in inducing autophagy, and subsequently, autophagy-induced cell death. This dual treatment has been found to be more effective in expressing autophagy-related protein and mRNA as well as increasing total autophagosomes. Furthermore, the treatment further decreases cell viability and increases apoptosis. We thus conclude that it may be beneficial to offer carbamazepine in conjunction with oncolytic reovirus in CRC patients with mutant *KRAS*.

## Supporting information

S1 Fig Raw blots from Fig 1. Raw unedited blots used in Fig 1.
(PDF)

S1 Table Primer Sequences. The forward and reverse primer sequences used for qPCR in Fig 2.
(XLSX)

S2 Table Effects of treatments on nucleus and chromatin area. Data regarding nucleus and chromatin area separated by treatment type. Lowest Chromatin/Nucleus ratio found in dual treatment group.
(XLSX)
