# Supplementary material for: Modulating autophagy in KRAS mutant colorectal cancer using combination of oncolytic reovirus and carbamazepine
Source: PLoS One. 2025 Jun 17;20(6):e0326029. doi: 10.1371/journal.pone.0326029 (PMC12173229; doi:10.1371/journal.pone.0326029)

6h - all blots

ATG5

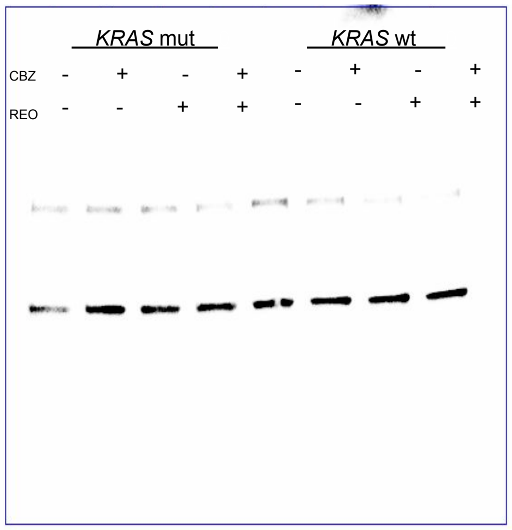

β-actin

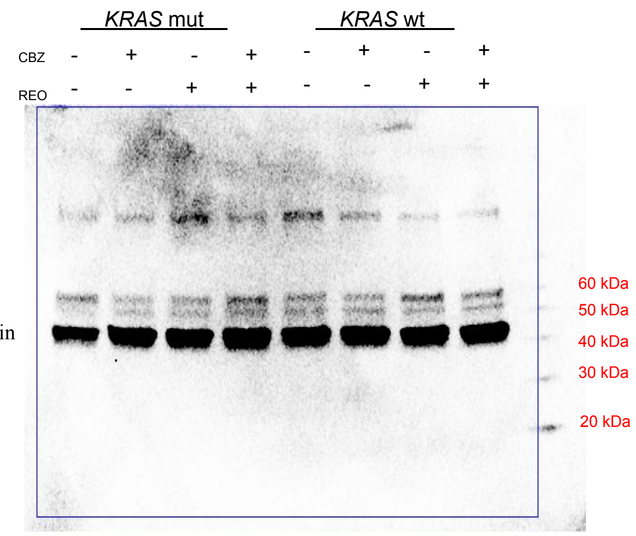

Beclin 1

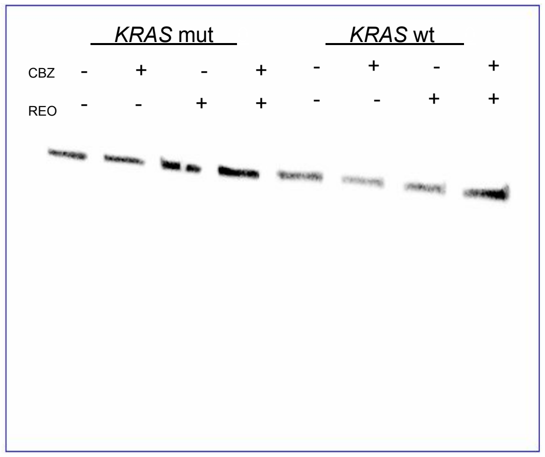

ULK1

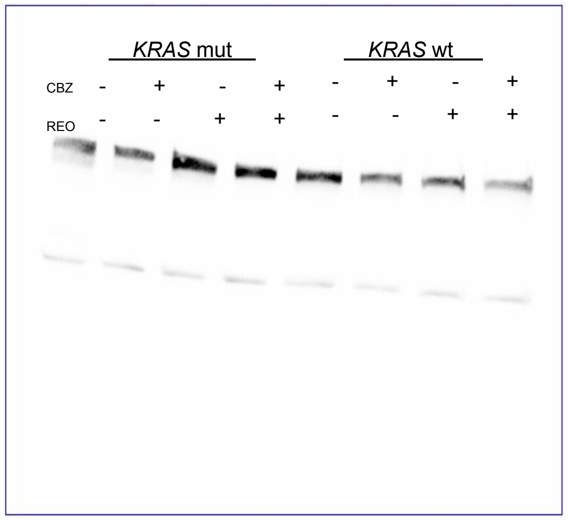

β-actin

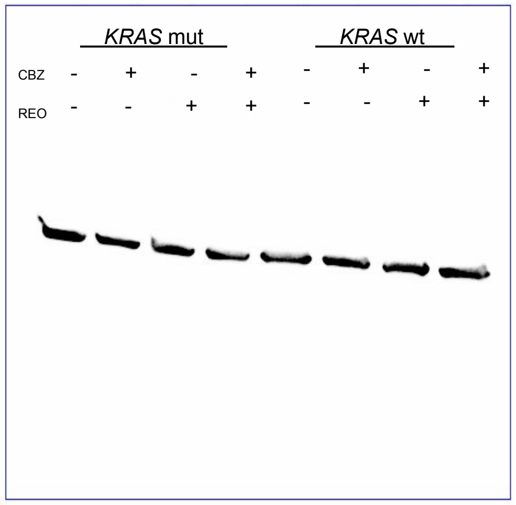

LC3B

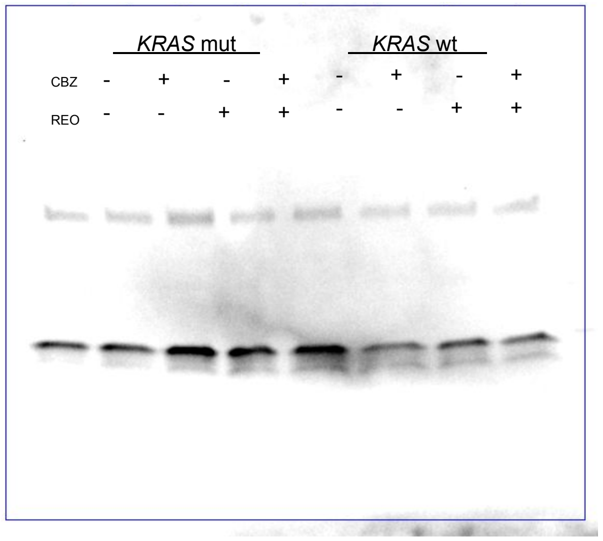

β-actin

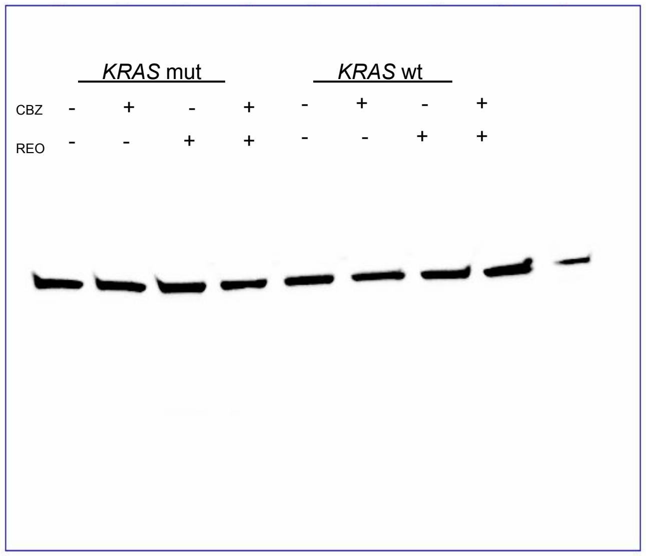

RICTOR

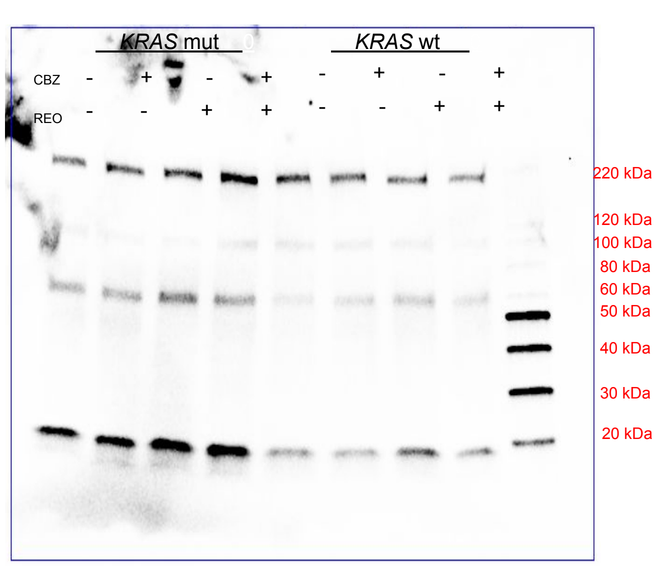

PIK3C3

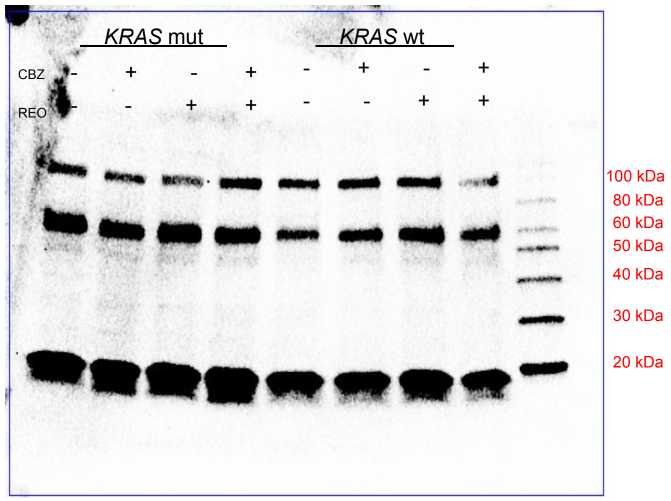

β-actin

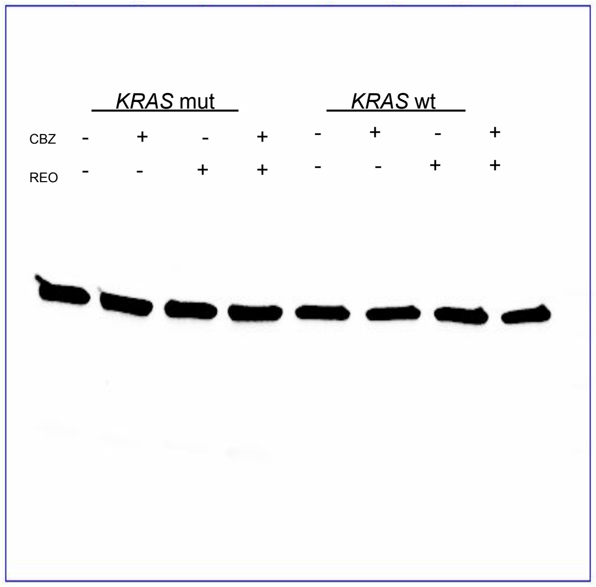

24h - all blots

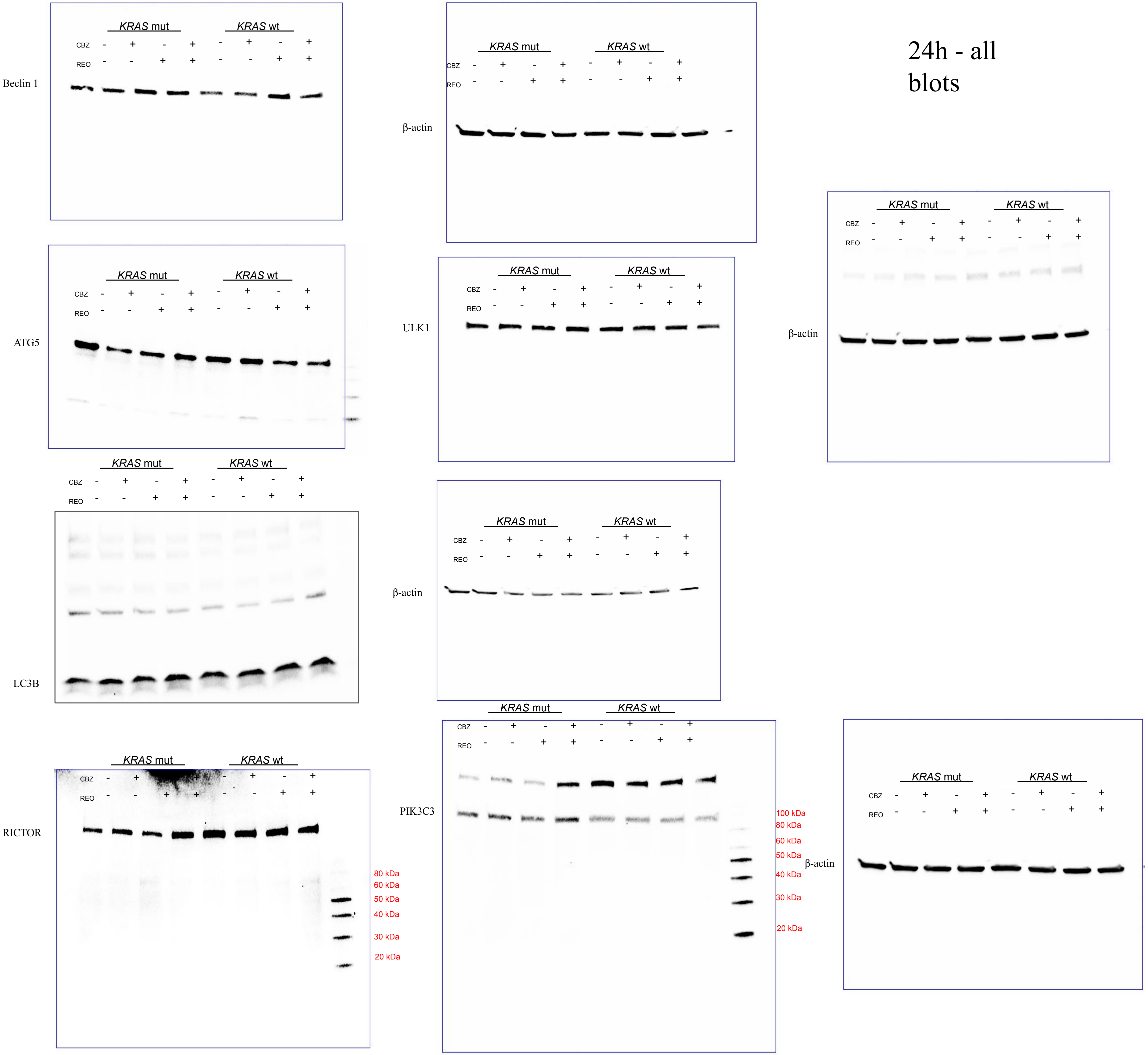

Supplement: S1 Fig — Raw unedited blots used in Fig 1. (PDF) [file pone.0326029.s001.pdf]
